# SLC41A1 overexpression correlates with immune cell infiltration in HCC and promotes its malignant progression

**DOI:** 10.7150/ijms.100155

**Published:** 2024-11-11

**Authors:** Gang Chen, Zhipeng Du, Caijun Rao

**Affiliations:** 1 Department of Geriatrics, Tongji Hospital, Tongji Medical College, Huazhong University of Science and Technology, Wuhan, China.; 2 Grade 2022 of Paediatrics, Second Clinical College, Tongji Hospital, Tongji Medical College, Huazhong University of Science and Technology, Wuhan, China.; 3 Department of Gastroenterology, Institute of Liver and Gastrointestinal Diseases, Tongji Hospital, Tongji Medical College, Huazhong University of Science and Technology, Wuhan, China.

**Keywords:** hepatocellular carcinoma, SLC41A1, prognosis, proliferation, migration, immune infiltration

## Abstract

Solute carrier 41 (SLC41) has been identified as a family of magnesium (Mg^2+^) transporters that participate in various diseases, including tumor development and progression. Recent studies revealed SLC41A3 acted as an oncogene and predicted poor survival for hepatocellular carcinoma (HCC) patients. However, the potential function of SLC41A1 in HCC remains unclear. In our study, we focused on the levels and putative mechanisms of SLC41A1 in HCC. Using bioinformatics techniques, we found SLC41A1 was upregulated in HCC, which was verified by immunostaining of HCC patients. SLC41A1 was correlated with clinicopathological characteristics, and could be utilized as independently diagnostic and prognostic markers for HCC. By exploring MethSurv website, DNA methylation was identified in SLC41A1, and several methylated CpG sites might affect overall survival of HCC patients. Using Gene Ontology (GO) , Kyoto Encyclopedia of Genes and Genomes (KEGG), gene set enrichment analysis (GSEA), protein-protein interaction (PPI) network, we found SLC41A1 overexpression was related to several tumor-promoting pathways and molecules, such as degradation of extracellular matrix, cell adhesion and O-linked glycosylation, and expression of CXCL1, CXCL5 and MUC1. The results of single-sample GSEA (ssGSEA) showed SLC41A1 might regulate infiltration of multiple immune cells, resulting in the imbalance between immune suppression and immune surveillance.

Cellular experiments showed that knockdown of SLC41A1 inhibited proliferation, migration and invasion of HCC, whereas SLC41A1 overexpression exerted the tumor-promoting effects. Collectively, our results shed light on new insights into expression, putative roles and mechanisms of SLC41A1 in HCC, providing novel diagnostic biomarkers and therapeutic targets for HCC.

## 1. Introduction

Liver cancer, which will account for over 830,000 fatalities worldwide in 2020, continues to rank third in the world for cancer-related mortality (GLOBOCAN 2020) 1. Much worse, the mortality is estimated to surpass 1,300,000 in 2040 2. The most prevalent type of primary liver cancer is hepatocellular carcinoma (HCC), accounting for ~90% of cases 3. The major curative methods for HCC include liver resection, ablation, radiotherapy, chemotherapy and liver transplantation, but these therapies require early diagnosis. Nevertheless, the majority of HCC patients receive a diagnosis at a fairly advanced stage 4, making these approaches relatively ineffective. Furthermore, high potential of recurrence or metastasis makes the five-year survival rate more dismal. Therefore, it is urgent to make further elucidation of the pathogenesis of HCC to develop novel therapeutic strategies.

There are three isoforms in the solute carrier 41 (SLC41) family: SLC41A1, SLC41A2, and SLC41A3. According to their distant homology with the magnesium (Mg^2+^) channel MgtE of bacteria, the SLC41 family were regarded as Mg^2+^ transporters, and played a critical part in maintaining Mg^2+^ homeostasis 5. SLC41A1 was identified as a sodium/magnesium (Na^+^/Mg^2+^) exchanger, and overexpression of SLC41A1 promoted Na^+^-dependent Mg^2+^ outflow across the plasma membrane 6. In the recent years, SLC41 family were reported to participate in various diseases, including tumor development and progression. Dysregulation and single nucleotide polymorphisms of SLC41A1 were related to Parkinson's disease 7, 8. Mutation of SLC41A1 disrupted renal Mg^2+^ homeostasis, led to tubular defects, and caused nephronophthisis-like phenotype 9. Furthermore, SLC41A1 was reported to be downregulated in human pancreatic ductal adenocarcinoma, and SLC41A1 overexpression inhibited cell proliferation and invasion, and suppressed orthotopic tumour growth in a mouse model 10. Recent studies reported SLC41A3 was upregulated in HCC, correlated with immune infiltration 11, and served as an unfavorable independent predictor of overall survival 12, 13. Knockdown of SLC41A3 impeded proliferation, migration and invasion of HCC cell lines 13. Nonetheless, the possible functions and putative mechanisms of SLC41A1 in HCC remain unclear.

In this study, by utilizing multiple bioinformatics tools, we comprehensively analyzed the expression and clinical relevance of SLC41A1 in HCC, evaluated their relationship to immune infiltration and DNA methylation, and investigated the functional enrichment, and assessed its role in HCC proliferation, migration and invasion. Our finding suggested that SLC41A1 was upregulated in HCC and predicted poor survival, SLC41A1 was correlated with DNA methylation and immune infiltration, and SLC41A1 promoted HCC proliferation, migration and invasion. Our study indicates that SLC41A1 might be valuable prognostic factor and therapeutic candidate in HCC.

## 2. Materials and methods

### 2.1 Analyses of SLC41A1 expression in public databases

50 normal and 374 tumor tissues are included in the online database The Cancer Genome Atlas (TCGA, https://portal.gdc.cancer.gov), which also contains clinical information and gene mRNA level of HCC patients. Gene expression profiles can be found in the Gene Expression Omnibus (GEO, https://www.ncbi.nlm.nih.gov/geo/) 14. The mRNA levels of SLC41A1 were analyzed in TCGA and two HCC GEO datasets (GSE25097 and GSE76427). The expression of SLC41A1 in the TCGA and GEO databases was examined by using the student t-test and the Wilcoxon rank sum test. As the clinical information for HCC cohort was derived from public database, written informed consent was supposed to have been already obtained.

### 2.2 The clinical values of SLC41A1 in HCC

In 50 normal and 374 tumor tissues from TCGA, patients were categorized into low expression and high expression groups based on the median mRNA level of SLC41A1 in TCGA HCC samples. The chi-square test was used to examine the connection between the expression of SLC41A1 and the clinicopathological parameters of HCC patients, such as gender, race, T, N, and M stages of the disease, histologic grade, etc. Utilizing Receiver Operating Curve (ROC) analysis, the diagnostic value of SLC41A1 in HCC was assessed. The prognostic value of SLC41A1 were assessed using COX regression analysis and Kaplan-Meier analysis (https://kmplot.com/analysis/).

### 2.3 Analysis of functional enrichment

Using the DESeq2 program, differentially expressed genes (DEGs) were found in patients with high and low SLC41A1 expression patterns 15. Genes having an adjusted P value less than 0.05 and an absolute fold change (FC) larger than 1.5 were subjected to additional analysis. DEGs associated with SLC41A1 were functionally annotated using the Gene Ontology (GO) enrichment analysis. Molecular functions (MF), cellular components (CC), and biological processes (BP) are all included in GO analysis. To clarify the pathways connected to the DEGs of SLC41A1, research was done using the Kyoto Encyclopedia of Genes and Genomes (KEGG). As for GO and KEGG analyses, adjusted P values less than 0.05 indicated significance 16. Using gene set enrichment analysis (GSEA), we investigated the biological pathways regulated by SLC41A1 overexpression 17. The enriched pathways were deemed statistically significant after 1000 permutations if the adjusted P values were less than 0.05 and the FDR was less than 0.25. The ClusterProfiler ggplot2 R software was utilized for statistical analysis and graphical plotting [3.3.6]. The Search Tool for the Retrieval of Interacting Genes (STRING) database was used to create the protein-protein interaction (PPI) network of DEGs associated with SLC41A1, and the R package igraph [1.4.1] was used to depict the top 20 PPI networks 18.

### 2.4 Cell culture

The HCC cell line SNU398 was purchased from the American Type Culture Collection (Manassas, USA), while Huh7 was obtained from Japanese Cancer Research Bank (Tokyo, Japan), and the two cell lines were preserved in our institute. SNU398 was cultured in RPMI-1640 Medium (GIBCO, USA) and Huh7 was cultivated in DMEM (GIBCO), all medium was supplemented with 10% fetal bovine serum (GIBCO) and 1% penicillin/streptomycin (Servicebio). Cells were cultured in a humidified atmosphere filled with 5% CO2 at 37 °C.

### 2.5 DNA methylation analysis

Using MethSurv (https://biit.cs.ut.ee/methsurv/), an interactive tool to assess prognosis based on DNA methylation state, we examined the DNA methylation sites of SLC41A1 and their predictive values in HCC.

### 2.6 Immune infiltration analysis

The gene set variation analysis (GSVA) package in R (4.2.1) was used to perform the single-sample gene set enrichment analysis (ssGSEA) in order to examine the relationship between SLC41A1 expression and the relative infiltration levels of 24 immune cells 19, and the correlation was determined by Spearman correlation analysis.

### 2.7 Immunohistochemistry (IHC) staining

The normal livers were collected from liver hemangioma patients, and the HCC and paracancerous non-tumor liver tissues (at least 2cm away from the tumor margin) were collected from HCC patients underwent surgery at Tongji Hospital, Tongji medical college, Huazhong University of Science and Technology (Wuhan, China) from June 2018 to December 2019. The liver tissues were fixed using 4% paraformaldehyde (Servicebio, Wuhan, China), embedded in paraffin, sectioned to 5µm thick sections. Detailed procedure of IHC staining was described previously 20. The primary antibody used in IHC is SLC41A1 (ab254754, Abcam, UK, 1:100). Written informed consent from patients was obtained, and this study approved by the Ethics Committee of Tongji Hospital and conducted following the Declaration of Helsinki.

### 2.8 Lentivirus transfection and stable cell line establishment

The SLC41A1 knocking-down (LV-shSLC41A1) and overexpressing lentiviruses (LV-SLC41A1) as well as the control lentiviruses (LV-shcontrol and LV-control) were purchased from Obio technology (Shanghai, China). The targeting sequences of shRNA are as follows, shRNA-1: 5'-GCTGCAAGTACTGTTCCCATT-3', shRNA-2: 5'-GATTGGGATCAACCCAGACAA-3'. The SNU398 were transfected with LV-shSLC41A1 and Huh7 were transfected with LV-SLC41A1 for 72 hours (h), and then the stable transfected cell line were established by puromycin selection for 14 days, as described previously 21. Subsequently, mRNA were extracted from these cells, and the quantative real-time PCR (qRT-PCR) was performed to measure the transfection efficiencies according to standard procedure 21. The primers used in qRT-PCR were listed below, SLC41A1, forward: 5'-CCAGAGCAACGAAAGTGACGA-3', reverse: 5'-TACTTGCAGCCCGATGGAAAA-3', GAPDH, forward: 5'-GCACCGTCAAGGCTGAGAAC-3', reverse: 5'-TGGTGAAGACGCCAGTGGA-3'.

### 2.9 Cell proliferation analyses

Cell proliferation was measured by Cell Counting Kit-8 (CCK-8) and 5-ethynyl-2'-deoxyuridine (EdU) assays, respectively. A number of 1000 indicated SNU398 and Huh7 cells were seeded into 96-well plates and then cultivated. As previously described 21, at each time point (24 h, 48 h, 72 h, 96 h), the cells were washed by PBS thrice and then incubated in a mixture containing 100 μl complete medium and 10 μl CCK-8 kit (Promoter Biotechnology, Wuhan, China) for 2h in the incubator. Finally, using a microplate reader (Biotek, Vermont, USA), the absorbance at 450 nm of cells were measured. The EdU assay was performed as previously described 21. Briefly, we plated 1×10^5^ SNU398 or Huh7 cells in 24-well plates, cultivated them for 24 h, and then added 10 mM EdU (Beyotime, Shanghai, China) solution to the wells and cultivated for another 2 h. The cell nuclei were counterstained by Hoechest 33342. The EdU-positive cells were observed using a fluorescence microscope (Olympus, Japan).

### 2.10 Cell migration and invasion analyses

Cell migration and invasion were measured by Transwell assays following procedures described previously 21. The Transwell chambers (8 μm pore size, Corning, USA) were pre-coated with Matrigel (BD Bioscience, USA) for invasion assay or not for migration assay, and then plated into 24-well plates. We re-suspended the indicated cells (5×10^4^ for migration and 1×10^5^ for invasion) with 200 μl serum-free DMEM, and then seeded them into the upper chamber, while 600 μl complete DMEM was added to the lower chamber for chemotaxis. Twenty-four hours later, the unmigrated cells on the upper surface of inserts were removed, and cell migrated across the membrane were fixed with 4% paraformaldehyde (Servicebio) for 20 min and then stained with crystal violet (Beyotime) for 1 h. Cells were observed and analyzed using a microscope (Olympus, Japan) in at least three fields of view.

### 2.11 Statistical Analysis

GraphPad Prism 5.01 (San Diego, CA, USA) and SPSS 19.0 (Chicago, IL, USA) were used for the statistical analysis. We used R (version 4.2.1) to conduct statistical analyses related to bioinformatics. P<0.05 was considered statistically significant.

## 3. Results

### 3.1 The expression, diagnostic value and prognostic value of SLC41A1 in HCC

**Figure 1:**
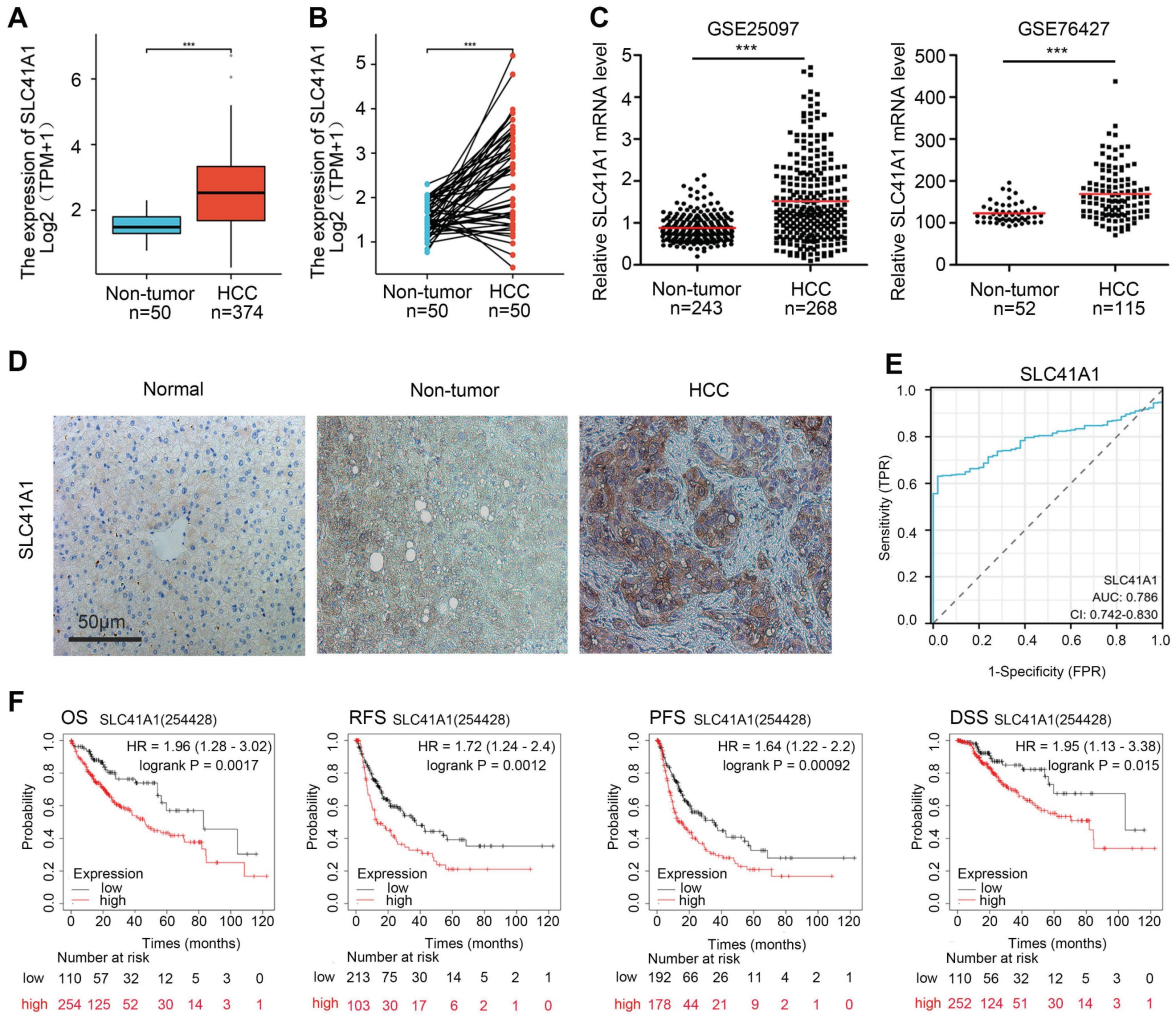
** The expression, diagnostic value and prognostic value of SLC41A1 in HCC.** (A-B) Relative mRNA expression of SLC41A1 in unpaired (A) and paired (B) HCC tissues in TCGA database. (C) Relative mRNA expression of SLC41A1 in unpaired non-tumor tissues and HCC tissues from datasets (GSE25097 and GSE76427) obtained from GEO dabatase. (D) Immunostaining of SLC41A1 in normal liver, paracancerous non-tumor tissues and HCC (n=6). (E) The ROC analysis of SLC41A1 in HCC. (F) Kaplan-Meier analysis showed prognostic value of SLC41A1 expression in predicting OS, RFS, PFS, DSS of HCC patients. ^***^P<0.001.

Firstly, we analyzed the SLC41A1 mRNA levels in HCC using the TCGA database. Compared to the non-tumor tissues, SLC41A1 expression was elevated in both unpaired HCC samples (1.5156±0.3675 vs 2.5438±1.1555) (Figure 1A) and paired HCC tissues (1.5156±0.3675 vs 2.5586±1.08) (Figure 1B). Furthermore, we explore the GEO datasets GSE25097 and GSE76427. We found that, compared with non-tumor samples, SLC41A1 was upregulated in HCC samples in GSE25097 (0.8817±0.3109 vs 1.5190±0.9653) and GSE76427 (123.0956±23.8637 vs 168.8677±62.4464) (Figure 1C). By immunostaining of HCC, non-tumor and normal liver sections, we confirmed the upregulation of SLC41A1 in HCC tissues compared to non-tumor and normal livers (Figure 1D). Secondly, we examined the relationship between clinicopathological characteristics and the expression of SLC41A1. We found that SLC41A1 was correlated with gender, tumor status, residual tumor, histologic grade (Table 1). Receiver operating characteristic (ROC) analysis revealed that the area under the curve (AUC) of SLC41A1 in normal and HCC tissues were 0.786 (Figure 1E), suggesting that SLC41A1 might be diagnostic markers for HCC. High expression of SLC41A1 is associated with shorter overall survival (OS), relapse-free survival (RFS), progression-free survival (PFS), and disease-specific survival (DSS), according to the Kaplan-Meier plotter survival analysis (Figure 1F). High SLC41A1 expression was an independent risk factor for the overall survival of HCC patients, according to the univariate and multivariate COX regression models (Table 2).

### 3.2 Prognostic value of DNA methylation of SLC41A1 in HCC

**Figure 2:**
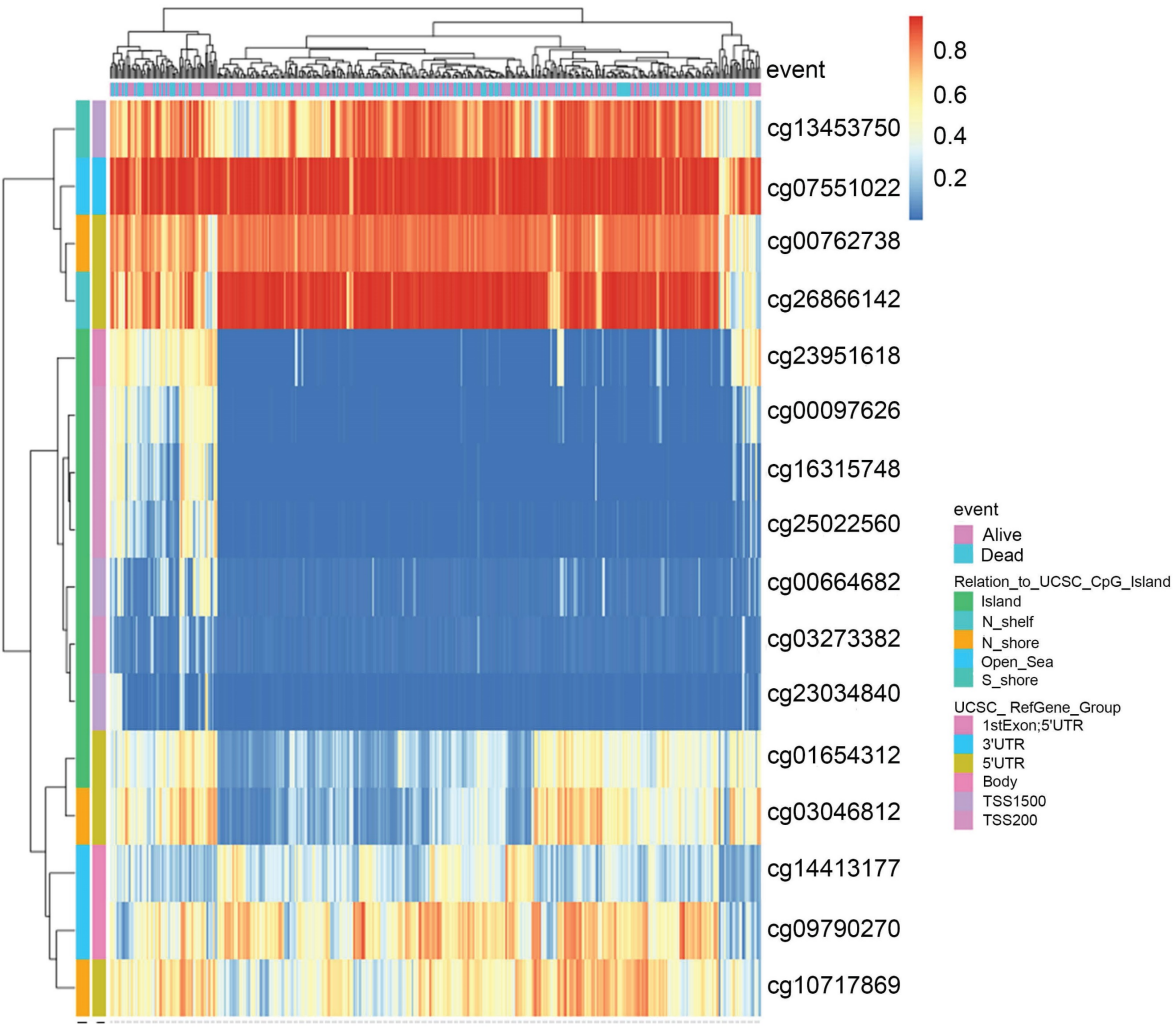
** The DNA methylation clustered expression of SLC41A1.** Sixteen CpG methylated sites in SLC41A1 were identified in MethSurv database, including cg13453750, cg07551022, cg00762738, cg26866142, cg23951618, cg00097626, cg16315748, cg25022560, cg00664682, cg03273382, cg23034840, cg01654312, cg03046812, cg14413177, cg09790270, cg10717869.

**Figure 3:**
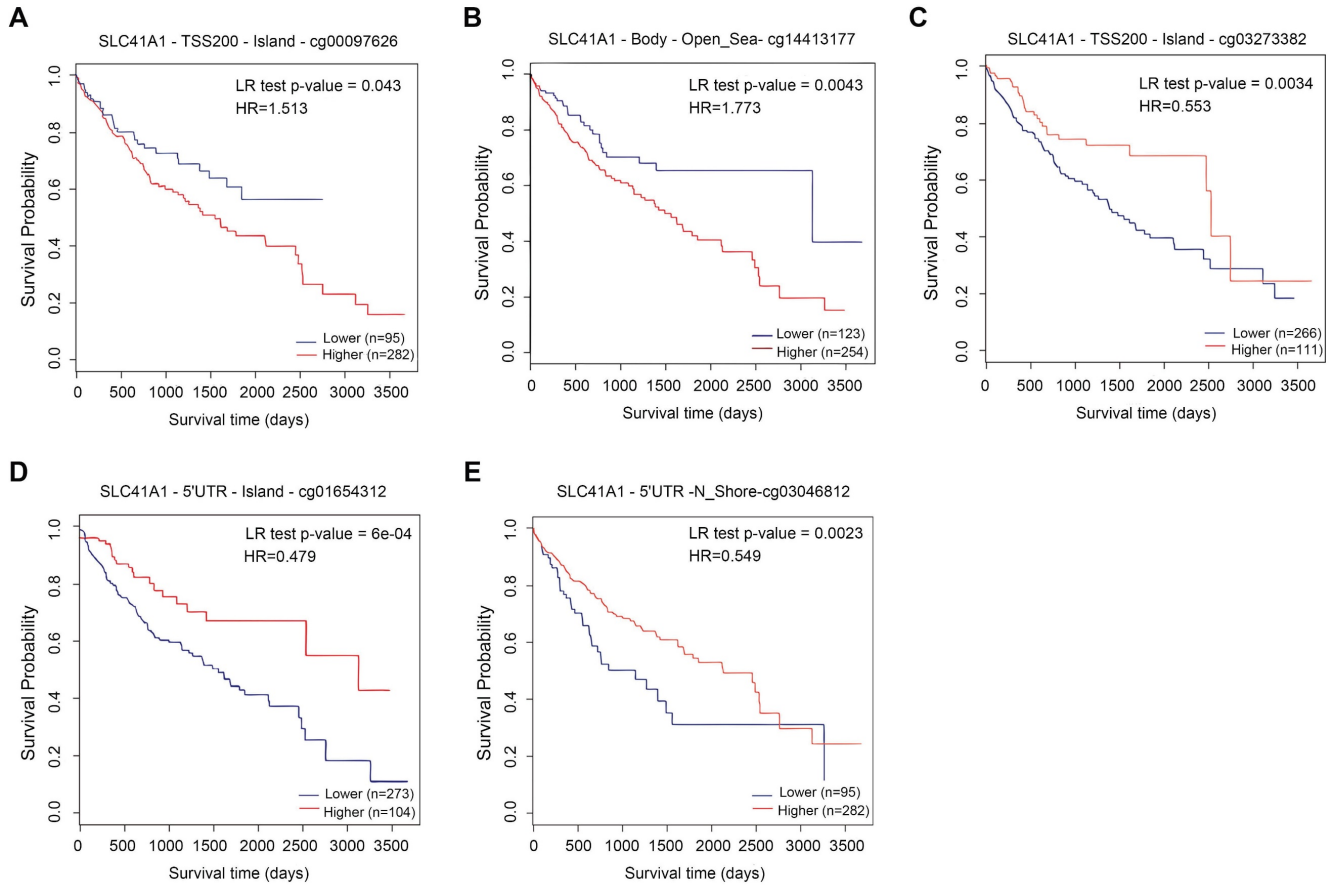
** Prognostic value of DNA methylation of SLC41A1 in HCC.** (A-E) Overall survival curve of SLC41A1 CpG methylated sites in HCC patients, including two unfavorable sites, cg00097626 (A), cg14413177 (B), and three favorable sites, cg03273382 (C), cg01654312 (D), and cg03046812 (E).

Recent studies have highlighted the role of DNA methylation biomarkers in HCC diagnosis and prognosis, revealing the mechanisms underlying HCC progression 22, 23. By analyzing the MethSurv database, we found that there existed sixteen CpG methylated sites in SLC41A1 (Figure 2). Further, among these CpG methylated sites, two sites were identified as unfavorable prognostic factors, including cg00097626 (HR=1.513, P=0.043) and cg14413177 (HR=1.773, P=0.0043), while three sites were identified as favorable prognostic factors, including cg03273382 (HR=0.553, P=0.0034), cg01654312 (HR=0.479, P=6e-04), cg03046812 (HR=0.549, P=0.0023) (Figure 3A-E).

### 3.3 Analysis of the functional enrichment of SLC41A1 expression in HCC

**Figure 4:**
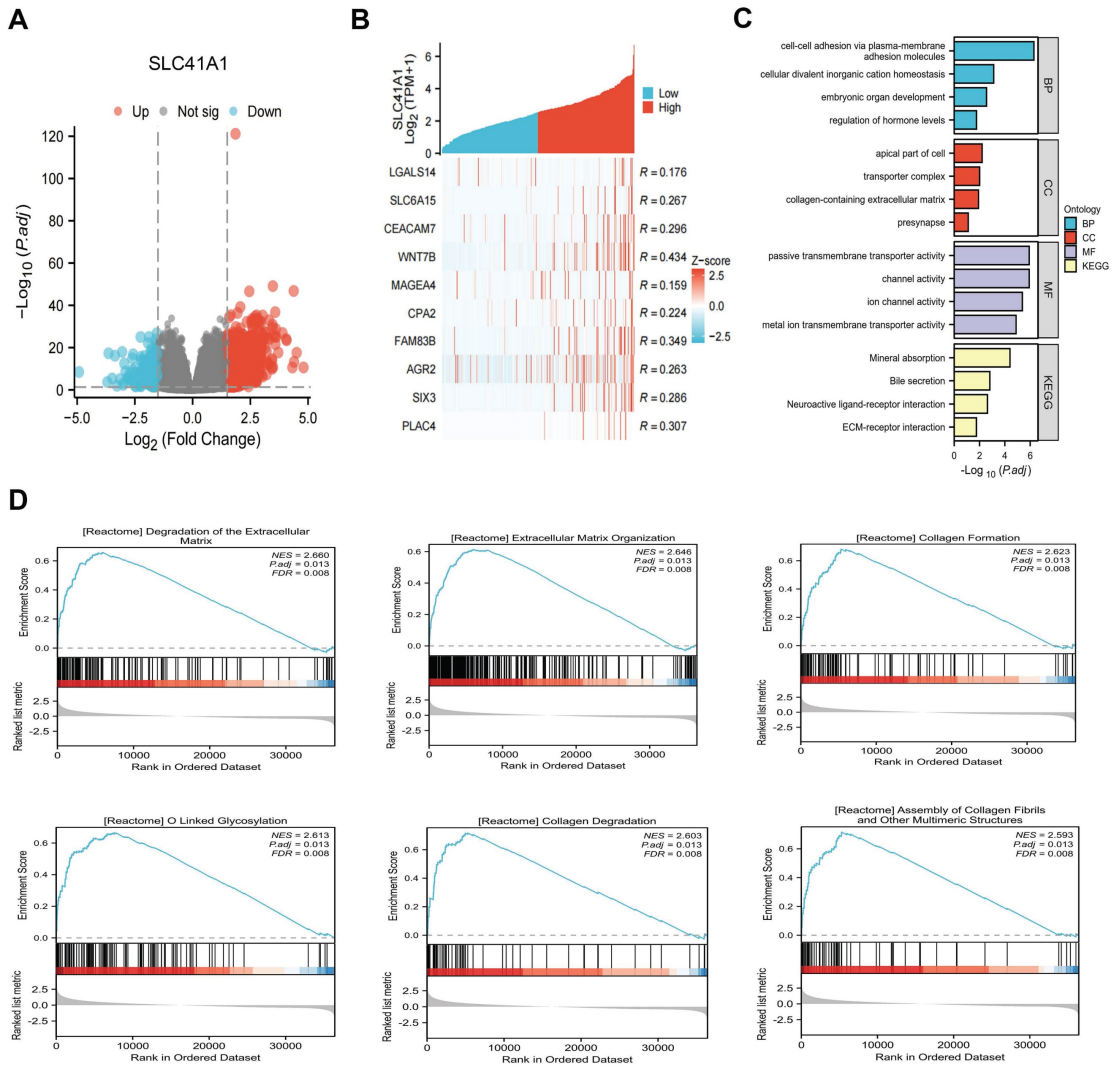
** Analysis of the functional enrichment of SLC41A1 expression in HCC.** (A) Volcano plot of the differentially expression genes (DEGs) relating to high/low expression of SLC41A1 in HCC from TCGA database. The DEGs were screened out based on |Log FC| > 1.5 and P<0.05. (B) Heatmap of top 10 upregulated DEGs of SLC41A1. (C) GO (BP, CC, MF) and KEGG analyses of DEGs of SLC41A1. (D) GSEA analysis of DEGs of SLC41A1, including degradation of extracellular matrix, extracellular matrix organization, collagen formation, O-linked glycosylation, collagen degradation and assembly of collagen fibrils and other multimeric structure.

**Figure 5:**
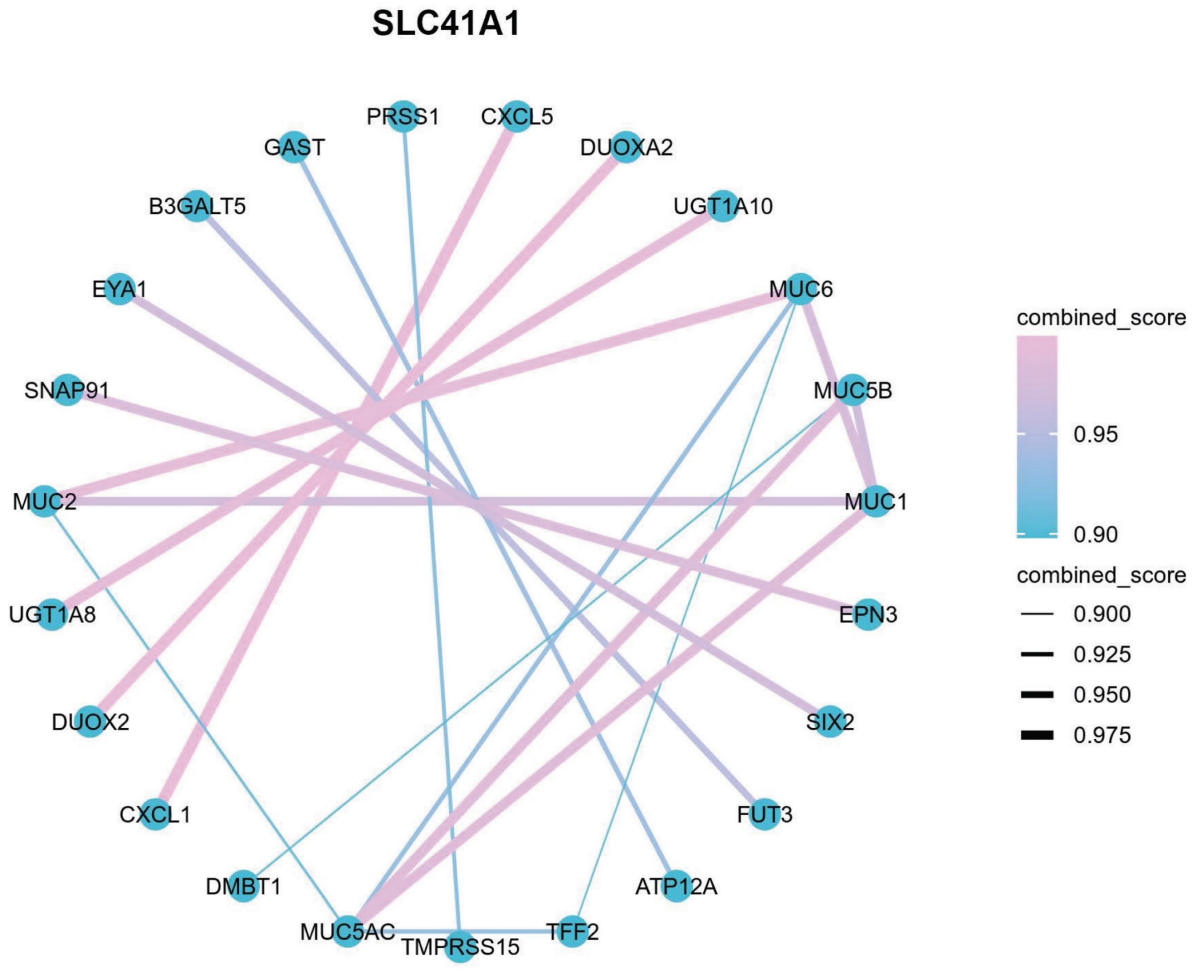
** PPI network (Top 20) of SLC41A1-associated genes.** Top 20 protein-protein interaction (PPI) network of SLC41A1-related genes were presented.

Using data from the TCGA cohort, we attempted to further clarify the possible mechanism of SLC41A1 in HCC. We classified the HCC patients into two groups: high expression and low expression, based on the expression levels of SLC41A1. By screening, we totally discovered 1039 SLC41A1-related differentially expressed genes (DEGs), of which 843 were upregulated and 196 decreased (Figure 4A). Gene expression heatmap was constructed to visualize the top 10 upregulated DEGs of SLC41A1 in HCC (Figure 4B). Further, we conducted GO and KEGG analyses, and found that SLC41A1 might regulate various biological processes, cellular components and molecular functions. SLC41A1 might be related to transporter complex, mediate metal ion chanel transporter activity, and thus regulate cellular divalent inorganic cation homeostasis. SLC41A1 might also regulate cell adhesion, collagen-containing extracellular matrix (ECM) and ECM-receptor interaction (Figure 4C). By GSEA, we further found that SLC41A1 was positively correlated with several tumor progression-related pathways, including degradation of ECM, ECM organization, O-linked glycosylation, collagen formation, collagen degradation and assembly of collagen fibrils (Figure 4D). In addition, the top 20 protein-protein interaction (PPI) network of SLC41A1-related genes were presented (Figure 5). The results showed that SLC41A1 was related to several oncogenes, such as CXCL1 24, CXCL5 25, MUC1 26, SIX2 27.

### 3.4 Correlation between immune infiltration and SLC41A1 expression in HCC

**Figure 6:**
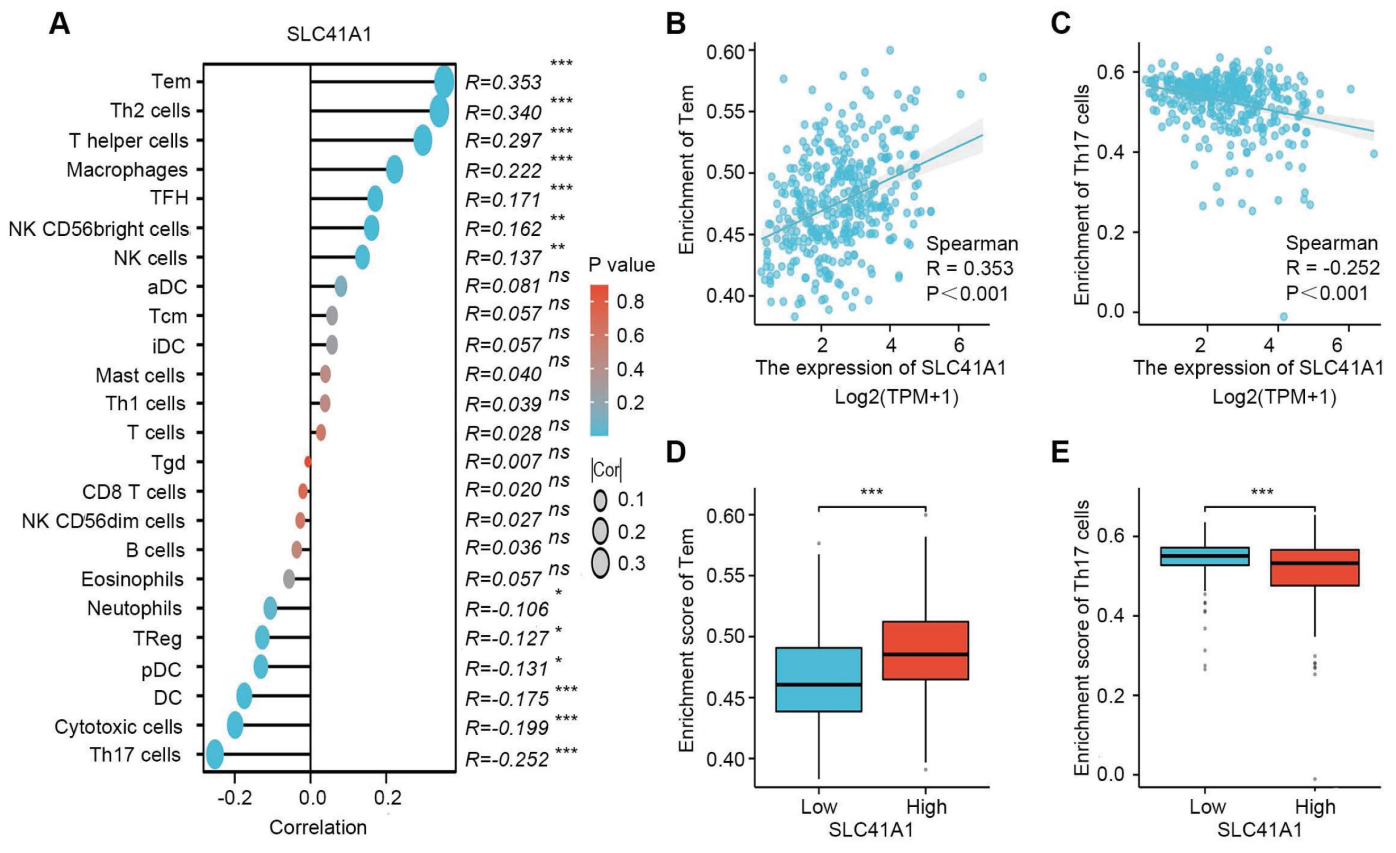
** Correlation between immune infiltration and SLC41A1 expression in HCC.** (A) SLC41A1 expression and the abundance of 24 types of immune cells. (B-C) Correlation between SLC41A1 expression and the abundance of Tem and Th17 cells. (D-E) Infiltration levels of Tem and Th17 cells in HCC patients with low or high SLC41A1 expression. ^*^P<0.05, ^* *^P<0.01, ^***^P<0.001.

The accumulation of various immune cells within the tumor microenvironment has been shown to contribute to HCC development and progression 28. Therefore, we further performed single-sample GSEA (ssGSEA) to explore the potential correlation between SLC41A1 expression and the infiltration levels of 24 types of immune cells. Our results showed that infiltration levels of effector memory T (Tem), T helper (Th) cells, Th2 cells, macrophages, follicular helper T (TFH), natural killer (NK) cells, NK CD56bright cells, were positively correlated with SLC41A1 expression, among which the correlation of Tem was the highest (R=0.353, P<0.001). On the contrary, infiltration levels of Th17 cells, cytotoxic cells, DC, pDC, Treg and neutrophils were negatively correlated with SLC41A1 expression, and the correlation of Th17 was the highest (R=-0.252, P<0.001) (Figure 6A-C). Furthermore, patients with elevated SLC41A1 expression levels showed reduced Th17 cell infiltration but increased Tem cell infiltration. (Figure 6D-E).

### 3.5 SLC41A1 promotes HCC cell proliferation, migration and invasion

**Figure 7:**
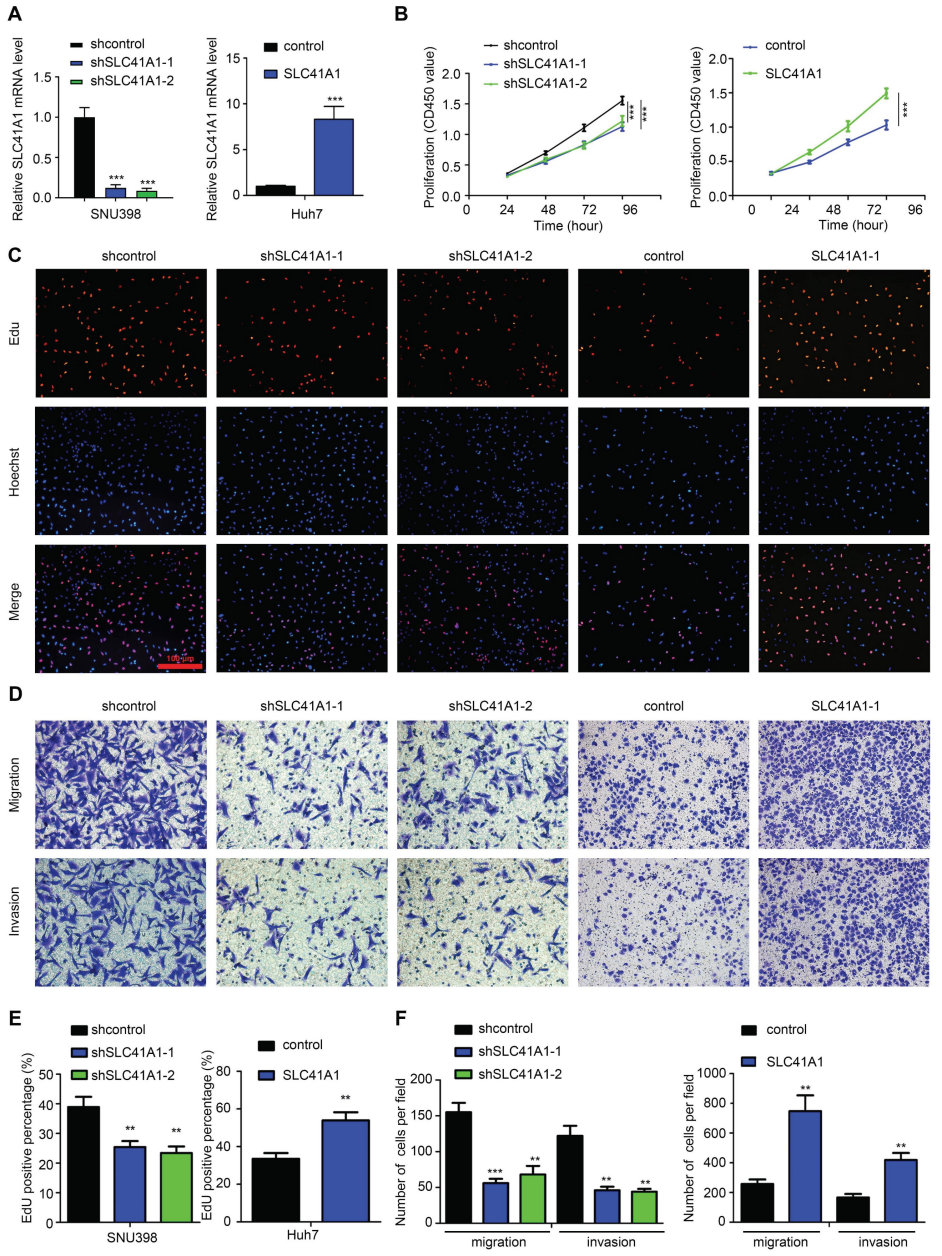
** SLC41A1 promotes HCC cell proliferation, migration and invasion.** (A) Transfection efficiencies of lentiviruses in SNU398 and Huh7 were measured by qRT-PCR. (B-C) The CCK-8 (B, n=6) and EdU (C, n=3) assays were conducted to assess cell proliferation. (D) The Transwell assays were performed to measure cell migration and invasion (n=3). (E)The EdU positive cell percent was shown. (F)The number of migrated and invaded cells was shown. ^**^P<0.01, ^***^P<0.001.

To verify the oncogenic role of SLC41A1 in HCC, we assessed the effect of SLC41A1 in HCC proliferation, migration and invasion. Firstly, we analyzed the relative mRNA expression of SLC41A1 in 24 kinds of HCC cell lines in Cancer Cell Line Encyclopedia (CCLE), the results were presented in Table 3. Considering the relative expression of SLC41A1 in HCC and the storage of HCC cell lines in our institute, we chose SNU398 (with relatively high expression of SLC41A1) and Huh7 (with relatively low expression of SLC41A1) for further exploration. The SNU398 cells were transfected with SLC41A1 knocking-down lentivirus and Huh7 cells were transfected with SLC41A1 overexpressing lentivirus. The results of RT-qPCR showed that the transfection efficiencies were satisfactory (Figure 7A). By CCK-8 analysis, we found that knockdown of SLC41A1 inhibited proliferation of SNU398 cells, while overexpression of SLC41A1 promoted proliferation of Huh7 cells (Figure 7B), and these effects were verified by EdU assay (Figure 7C, E). Furthermore, the results of Transwell assays demonstrated that knockdown of SLC41A1 inhibited migration and invasion of SNU398, whereas SLC41A1 overexpression facilitated Huh7 migration and invasion (Figure 7D, F).

## 4. Discussion

In the past three decades, the world has been suffering from HCC-related deaths. It is estimated that approximately 1 million people are expected to be affected by liver cancer by 2025 3. Despite advances have been achieved in the field of HCC diagnosis and treatment, most HCC patients are diagnosed at advanced stage and the prognosis is still unsatisfactory. Thus, uncovering novel target for diagnosis and treatment of HCC is urgently needed.

In the organism, Mg^2+^ is a vital ion that plays prominent roles in many cellular processes, and participates in immune regulation, inflammation, oxidative stress, cell cycle progression, differentiation, and apoptosis, and exerts crucial roles in development of progression of tumors 29. As a family of Mg^2+^ transporter, the SLC41 family consisted of three members, and SLC41A3 was identified as an oncogene and predicted poor survival for HCC by several studies 11-13.

In this study, we revealed that SLC41A1 is an oncogene in HCC. Data from TCGA and GEO database consistently showed that SLC41A1 was upregulated in HCC tissues, compared to non-tumor tissues. By immunostaining, we verified that expression of SLC41A1 was dramatically upregulated in HCC tissues than in non-tumor tissues and normal liver tissues, which made our results more convincing. Clinically, aberrant expression of SLC41A1 were correlated with clinicopathological characteristics, demonstrating that high SLC41A1 expression was positively associated with several unfavorable factors, such as tumor status, residual tumor and histologic grade. Furthermore, high SLC41A1 expression were correlated with poorer OS, RFS, PFS, DSS, respectively. These results suggested that, similar to SLC41A3, SLC41A1 acted as an independently unfavorable diagnostic and prognostic factor in HCC.

As a major form of epigenetic modification, DNA methylation is responsible for gene transcription, and is reported to influence HCC development, progression and prognosis 22, 23. The bioinformatics results predicted that SLC41A1 genes harbored several methylated CpG sites in HCC database, which might be responsible for the aberrant expression of SLC41A1. Furthermore, we found methylation of different CpG sites might be related to poorer or better overall survival of HCC patients.

To further explore the function of SLC41A1 in HCC, we conducted GO, KEGG, GSEA and PPI analyses. The GO items, including 'cellular divalent inorganic cation homeostasis', 'transporter complex', 'metal ion transmembrane transporter activity' might all indicate the role of SLC41A1 in Mg^2+^ transport. Furthermore, the GO items 'cell-cell adhesion via plasma-membrane adhesion molecules', 'collagen-containing extracellular matrix', and the KEGG item 'ECM receptor interaction' suggested SLC41A1 mediated function of ECM. The GSEA results showed SLC41A1 is related to several tumor-promoting signaling, such as extracellular matrix degradation, collagen degradation, O linked glycosylation, and these signaling contributed to HCC progression 30, 31. The PPI network of also showed SLC41A was related to several tumor-promoting genes, such as CXCL1, CXCL5 and MUC1, overexpression of these genes promoted HCC cell growth and invasion 25, 26, 32. The bioinformatics results suggested that SLC41A1 might function as an oncogene through these pathways.

With the rapid development and application of immune checkpoint inhibitors, HCC therapy is now entering the era of biologics. In this study, we explored the correlation between infiltration levels of 24 immune cells and the expression of SLC41A1. Our result showed that SLC41A1 expression was associated with infiltration of several immune cells, especially Tem and Th17 cells, suggesting that SLC41A1 might regulate the immune homeostasis of HCC. However, the exact effects of SLC41A1 on immune infiltration in HCC need further experimental investigation.

Furthermore, we assessed the role of SLC41A1 in HCC by cellular experiments. To ensure the credibility of the results, we chose SNU398 and Huh7 for genetic manipulation according to the baseline expression (CCLE). By loss- and gain- of function assays, our results showed that SLC41A1 could exert its oncogenic role by promoting proliferation, migration and invasion of HCC. Similarly, SLC41A1 expression was increased in breast cancer, and knockdown of SLC41A1 decreased cell viability in breast cancer cells 33. However, the roles of SLC41A1 in HCC was opposite to those in human pancreatic ductal adenocarcinoma, where SLC41A1 was downregulated and inhibited proliferation, migration and invasion through magnesium-dependent Akt/mTOR inhibition and bax-regulated apoptosis 10. Combining our data and the previous reports, we can draw the conclusion that different expression pattern of SLC41A1 indicates specific roles in certain diseases. These discrepancies might be derived from different genetic background caused by tissue specificity.

Despite our findings, this study has some limitations. Firstly, we speculate several candidate molecules and signaling pathways that might underlie the mechanisms of SLC41A1 in HCC by bioinformatics, which need further validation by experiments. Secondly, we assessed the role of SLC41A1 in HCC by *in vitro* experiments, however, these effects will be more convincing if validated by *in vivo* research. These assays will be implemented in our future studies.

Collectively, our results provided new insights into expression, putative roles and mechanism of SLC41A1 in HCC, providing a novel diagnostic biomarker and therapeutic target for HCC.

## Figures and Tables

**Table 1:** The association between SLC41A1 expression and clinicopathological characteristics.

Characteristics	Low expression of SLC41A1	High expression of SLC41A1	P value
n	187	187	
Gender, n (%)			**0.020**
Female	50 (13.4%)	71 (19%)	
Male	137 (36.6%)	116 (31%)	
Race, n (%)			0.728
Asian	82 (22.7%)	78 (21.5%)	
Black or African American	10 (2.8%)	7 (1.9%)	
White	91 (25.1%)	94 (26%)	
Age, n (%)			0.377
<= 60	84 (22.5%)	93 (24.9%)	
> 60	102 (27.3%)	94 (25.2%)	
Height, n (%)			0.693
< 170	102 (29.9%)	99 (29%)	
>= 170	68 (19.9%)	72 (21.1%)	
Weight, n (%)			0.518
<= 70	89 (25.7%)	95 (27.5%)	
> 70	84 (24.3%)	78 (22.5%)	
BMI, n (%)			0.209
<= 25	83 (24.6%)	94 (27.9%)	
> 25	86 (25.5%)	74 (22%)	
Pathologic T stage, n (%)			0.160
T1	102 (27.5%)	81 (21.8%)	
T2	42 (11.3%)	53 (14.3%)	
T3	36 (9.7%)	44 (11.9%)	
T4	5 (1.3%)	8 (2.2%)	
Pathologic N stage, n (%)			0.139
N0	127 (49.2%)	127 (49.2%)	
N1	0 (0%)	4 (1.6%)	
Pathologic M stage, n (%)			1.000
M0	135 (49.6%)	133 (48.9%)	
M1	2 (0.7%)	2 (0.7%)	
Pathologic stage, n (%)			0.197
Stage I	98 (28%)	75 (21.4%)	
Stage II	40 (11.4%)	47 (13.4%)	
Stage III	38 (10.9%)	47 (13.4%)	
Stage IV	2 (0.6%)	3 (0.9%)	
Tumor status, n (%)			**0.037**
Tumor free	111 (31.3%)	91 (25.6%)	
With tumor	67 (18.9%)	86 (24.2%)	
Residual tumor, n (%)			**0.025**
R0	170 (49.3%)	157 (45.5%)	
R1	4 (1.2%)	13 (3.8%)	
R2	1 (0.3%)	0 (0%)	
Histologic grade, n (%)			**0.038**
G1	37 (10%)	18 (4.9%)	
G2	89 (24.1%)	89 (24.1%)	
G3	55 (14.9%)	69 (18.7%)	
G4	5 (1.4%)	7 (1.9%)	
Child-Pugh grade, n (%)			0.805
A	119 (49.4%)	100 (41.5%)	
B	13 (5.4%)	8 (3.3%)	
C	1 (0.4%)	0 (0%)	
Fibrosis ishak score, n (%)			0.963
0	39 (18.1%)	36 (16.7%)	
1/2	16 (7.4%)	15 (7%)	
3/4	16 (7.4%)	12 (5.6%)	
5&6	44 (20.5%)	37 (17.2%)	
Adjacent hepatic tissue inflammation, n (%)			0.075
None	63 (26.6%)	55 (23.2%)	
Mild	45 (19%)	56 (23.6%)	
Severe	13 (5.5%)	5 (2.1%)	
Vascular invasion, n (%)			0.112
No	114 (35.8%)	94 (29.6%)	
Yes	50 (15.7%)	60 (18.9%)	
Albumin(g/dl), n (%)			0.200
< 3.5	41 (13.7%)	28 (9.3%)	
>= 3.5	117 (39%)	114 (38%)	
AFP(ng/ml), n (%)			0.109
<= 400	117 (41.8%)	98 (35%)	
> 400	28 (10%)	37 (13.2%)	

**Table 2:** Univariate and multivariate COX regression analyses revealed the risk factors for OS in HCC patients.

Characteristics	Total(N)	Univariate analysis			Multivariate analysis	
		Hazard ratio (95% CI)	P value		Hazard ratio (95% CI)	P value
Gender	373					
Female	121	Reference				
Male	252	0.793 (0.557 - 1.130)	0.200			
Race	361					
Asian	159	Reference				
Black or African American & White	202	1.341 (0.926 - 1.942)	0.121			
Age	373					
<= 60	177	Reference				
> 60	196	1.205 (0.850 - 1.708)	0.295			
Height	340					
< 170	201	Reference				
>= 170	139	1.232 (0.849 - 1.788)	0.272			
BMI	336					
<= 25	177	Reference				
> 25	159	0.798 (0.550 - 1.158)	0.235			
Pathologic T stage	370					
T1&T2	277	Reference			Reference	
T3&T4	93	2.598 (1.826 - 3.697)	**< 0.001**		2.147 (1.227 - 3.756)	**0.007**
Pathologic N stage	258					
N0	254	Reference				
N1	4	2.029 (0.497 - 8.281)	0.324			
Pathologic M stage	272					
M0	268	Reference			Reference	
M1	4	4.077 (1.281 - 12.973)	**0.017**		0.960 (0.225 - 4.092)	0.956
Pathologic stage	349					
Stage I	173	Reference			Reference	
Stage II&Stage III&Stage IV	176	2.090 (1.429 - 3.055)	**< 0.001**		1.450 (0.791 - 2.659)	0.230
Tumor status	354					
Tumor free	202	Reference			Reference	
With tumor	152	2.317 (1.590 - 3.376)	**< 0.001**		1.980 (1.238 - 3.167)	**0.004**
Residual tumor	344					
R0	326	Reference				
R1&R2	18	1.604 (0.812 - 3.169)	0.174			
Histologic grade	368					
G1&G2	233	Reference				
G3&G4	135	1.091 (0.761 - 1.564)	0.636			
Weight	345					
<= 70	184	Reference				
> 70	161	0.941 (0.657 - 1.346)	0.738			
Child-Pugh grade	240					
A	218	Reference				
B&C	22	1.643 (0.811 - 3.330)	0.168			
Fibrosis ishak score	214					
0&1/2	106	Reference				
3/4&5&6	108	0.740 (0.445 - 1.232)	0.247			
Adjacent hepatic tissue inflammation	236					
None	118	Reference				
Mild&Severe	118	1.194 (0.734 - 1.942)	0.475			
Vascular invasion	317					
No	208	Reference				
Yes	109	1.344 (0.887 - 2.035)	0.163			
AFP(ng/ml)	279					
<= 400	215	Reference				
> 400	64	1.075 (0.658 - 1.759)	0.772			
SLC41A1	373					
Low	186	Reference			Reference	
High	187	1.741 (1.225 - 2.474)	**0.002**		1.797 (1.140 - 2.832)	**0.012**

**Table 3:** Relative mRNA expression of SLC41A1 in HCC cell lines (CCLE).

HCC	SLC41A1
HEP3B217	1.646162657
JHH5	1.963474124
JHH4	3.375734539
**HUH7**	3.486714373
JHH7	3.503348735
HUH1	3.624100895
SNU761	4.057450272
JHH2	4.08236197
JHH1	4.169123281
PLCPRF5	4.175524601
KMCH1	4.320484678
LI7	4.460742564
SNU886	4.512226887
SKHEP1	4.628773595
JHH6	4.705425039
SNU387	4.718635616
SNU449	4.771357409
SNU182	4.808899839
**SNU398**	4.892391026
SNU878	4.921721947
SNU739	5.09085343
SNU423	5.194559886
HLF	5.38024459
SNU475	5.448900951

## References

[1] Sung H, Ferlay J, Siegel RL, Laversanne M, Soerjomataram I, Jemal A (2021). Global Cancer Statistics 2020: GLOBOCAN Estimates of Incidence and Mortality Worldwide for 36 Cancers in 185 Countries. CA Cancer J Clin.

[2] Rumgay H, Arnold M, Ferlay J, Lesi O, Cabasag CJ, Vignat J (2022). Global burden of primary liver cancer in 2020 and predictions to 2040. J Hepatol.

[3] Llovet JM, Kelley RK, Villanueva A, Singal AG, Pikarsky E, Roayaie S (2021). Hepatocellular carcinoma. Nat Rev Dis Primers.

[4] D'Amico G, Morabito A, D'Amico M, Pasta L, Malizia G, Rebora P (2018). Clinical states of cirrhosis and competing risks. J Hepatol.

[5] Nemoto T, Tagashira H, Kita T, Kita S, Iwamoto T (2023). Functional characteristics and therapeutic potential of SLC41 transporters. J Pharmacol Sci.

[6] Kolisek M, Nestler A, Vormann J, Schweigel-Rontgen M (2012). Human gene SLC41A1 encodes for the Na+/Mg(2)+ exchanger. Am J Physiol Cell Physiol.

[7] Lin L, Yan M, Wu B, Lin R, Zheng Z (2018). Expression of magnesium transporter SLC41A1 in the striatum of 6-hydroxydopamine-induced parkinsonian rats. Brain Res Bull.

[8] Cibulka M, Brodnanova M, Grendar M, Necpal J, Benetin J, Han V (2022). Alzheimer's Disease-Associated SNP rs708727 in SLC41A1 May Increase Risk for Parkinson's Disease: Report from Enlarged Slovak Study. Int J Mol Sci.

[9] Hurd TW, Otto EA, Mishima E, Gee HY, Inoue H, Inazu M (2013). Mutation of the Mg2+ transporter SLC41A1 results in a nephronophthisis-like phenotype. J Am Soc Nephrol.

[10] Xie J, Cheng CS, Zhu XY, Shen YH, Song LB, Chen H (2019). Magnesium transporter protein solute carrier family 41 member 1 suppresses human pancreatic ductal adenocarcinoma through magnesium-dependent Akt/mTOR inhibition and bax-associated mitochondrial apoptosis. Aging (Albany NY).

[11] Liu J, Zhang S, Dai W, Xie C, Li JC (2020). A Comprehensive Prognostic and Immune Analysis of SLC41A3 in Pan-Cancer. Front Oncol.

[12] Li Q, Xiong DL, Wang H, Jin WL, Ma YY, Fan XM (2021). High Expression of SLC41A3 Correlates with Poor Prognosis in Hepatocellular Carcinoma. Onco Targets Ther.

[13] Chang Q, Xu Y, Wang J, Jing H, Rao L, Tang W (2021). SLC41A3 Exhibits as a Carcinoma Biomarker and Promoter in Liver Hepatocellular Carcinoma. Comput Math Methods Med.

[14] Barrett T, Wilhite SE, Ledoux P, Evangelista C, Kim IF, Tomashevsky M (2013). NCBI GEO: archive for functional genomics data sets-update. Nucleic Acids Res.

[15] Love MI, Huber W, Anders S (2014). Moderated estimation of fold change and dispersion for RNA-seq data with DESeq2. Genome Biol.

[16] Yu G, Wang LG, Han Y, He QY (2012). clusterProfiler: an R package for comparing biological themes among gene clusters. OMICS.

[17] Subramanian A, Tamayo P, Mootha VK, Mukherjee S, Ebert BL, Gillette MA (2005). Gene set enrichment analysis: a knowledge-based approach for interpreting genome-wide expression profiles. Proc Natl Acad Sci U S A.

[18] Szklarczyk D, Gable AL, Lyon D, Junge A, Wyder S, Huerta-Cepas J (2019). STRING v11: protein-protein association networks with increased coverage, supporting functional discovery in genome-wide experimental datasets. Nucleic Acids Res.

[19] Hanzelmann S, Castelo R, Guinney J (2013). GSVA: gene set variation analysis for microarray and RNA-seq data. BMC Bioinformatics.

[20] Du Z, Lin Z, Wang Z, Liu D, Tian D, Xia L (2020). SPOCK1 overexpression induced by platelet-derived growth factor-BB promotes hepatic stellate cell activation and liver fibrosis through the integrin alpha5beta1/PI3K/Akt signaling pathway. Lab Invest.

[21] Du Z, Zhang Z, Han X, Xie H, Yan W, Tian D (2023). Comprehensive Analysis of Sideroflexin 4 in Hepatocellular Carcinoma by Bioinformatics and Experiments. Int J Med Sci.

[22] Nagaraju GP, Dariya B, Kasa P, Peela S, El-Rayes BF (2022). Epigenetics in hepatocellular carcinoma. Semin Cancer Biol.

[23] Fu S, Debes JD, Boonstra A (2023). DNA methylation markers in the detection of hepatocellular carcinoma. Eur J Cancer.

[24] Li L, Xu L, Yan J, Zhen ZJ, Ji Y, Liu CQ (2015). CXCR2-CXCL1 axis is correlated with neutrophil infiltration and predicts a poor prognosis in hepatocellular carcinoma. J Exp Clin Cancer Res.

[25] Zhou SL, Dai Z, Zhou ZJ, Wang XY, Yang GH, Wang Z (2012). Overexpression of CXCL5 mediates neutrophil infiltration and indicates poor prognosis for hepatocellular carcinoma. Hepatology.

[26] Wang J, Ni WH, Hu KB, Zhai XY, Xie F, Jie J (2017). Targeting MUC1 and JNK by RNA interference and inhibitor inhibit the development of hepatocellular carcinoma. Cancer Sci.

[27] Wan ZH, Ma YH, Jiang TY, Lin YK, Shi YY, Tan YX (2019). Six2 is negatively correlated with prognosis and facilitates epithelial-mesenchymal transition via TGF-beta/Smad signal pathway in hepatocellular carcinoma. Hepatobiliary Pancreat Dis Int.

[28] Hou J, Zhang H, Sun B, Karin M (2020). The immunobiology of hepatocellular carcinoma in humans and mice: Basic concepts and therapeutic implications. J Hepatol.

[29] Ashique S, Kumar S, Hussain A, Mishra N, Garg A, Gowda BHJ (2023). A narrative review on the role of magnesium in immune regulation, inflammation, infectious diseases, and cancer. J Health Popul Nutr.

[30] Desert R, Chen W, Ge X, Viel R, Han H, Athavale D (2023). Hepatocellular carcinomas, exhibiting intratumor fibrosis, express cancer-specific extracellular matrix remodeling and WNT/TGFB signatures, associated with poor outcome. Hepatology.

[31] Zhang J, Xun M, Li C, Chen Y (2022). The O-GlcNAcylation and its promotion to hepatocellular carcinoma. Biochim Biophys Acta Rev Cancer.

[32] Zhao H, Wei S, Zhou D, Liu Y, Guo Z, Fang C (2023). Blocking the CXCL1-CXCR2 axis enhances the effects of doxorubicin in HCC by remodelling the tumour microenvironment via the NF-kappaB/IL-1beta/CXCL1 signalling pathway. Cell Death Discov.

[33] Uddin MB, Balaravi Pillai B, Tha KK, Ashaie M, Karim ME, Chowdhury EH (2018). Carbonate Apatite Nanoparticles-Facilitated Intracellular Delivery of siRNA(s) Targeting Calcium Ion Channels Efficiently Kills Breast Cancer Cells. Toxics.

